# Coaches’ Perceptions of Factors Driving Training Adaptation: An International Survey

**DOI:** 10.1007/s40279-023-01894-1

**Published:** 2023-08-08

**Authors:** Kechi Anyadike-Danes, Lars Donath, John Kiely

**Affiliations:** 1 Department of Intervention Research in Exercise Training, German Sport University Cologne, Cologne, Germany; 2 Faculty of Education and Health Sciences, University of Limerick, Limerick, Ireland

## Abstract

**Objective:**

We surveyed coaches’ views on topics related to the training process to elucidate whether their opinions are aligned with the current literature. Here the results for a sub-set of questions regarding factors affecting the training adaptation process are presented and discussed.

**Methods:**

106 coaches [age range 18–65 + years, 31% 15 + years coaching, 58% individual-events/sports and 32% international level] from a number of countries completed a novel cross-sectional online survey about the planning of training and the training process.

**Results:**

Only 28% of participants indicated that physical training was the most important factor in determining sport performance; whereas 99% indicated non-physical factors influence physical training response. The top five factors in modifying an athlete’s ability to physically adapt to a training plan, as rated ‘absolutely essential’, were ‘coach-athlete relationship’ (56%), ‘life stress’ (41%), ‘athletes’ belief in the plan’ (37%), ‘psychological and emotional stress’ (35%) and ‘physical training’ (33%).

**Conclusions:**

Amongst coaches surveyed less than a third rated physical training as the most important factor in determining sports performance. Non-physical factors were acknowledged by the majority to exert an influence on physical training response and adaptation, despite the lack of discussion in training research, though there was no consensus on the relative importance of each individual factor. We echo previous sentiments that coaches need to be engaged in the research process. If training research continues as present the field runs the risk of not only becoming detached but increasingly irrelevant to those it is trying to help.

**Supplementary Information:**

The online version contains supplementary material available at 10.1007/s40279-023-01894-1.

## Key Points

**Table:** 

Coaches viewed non-physical factors as playing an important role in influencing the response to physical training.
Four non-physical factors considered by the coaches to be important in determining how an athlete will physically adapt to a training plan are rarely if ever acknowledged in the training literature.
There is a scientific basis for these factors playing an important role, as evidenced by research, leading training theory to be slightly at odds with both coaches’ views and scientific evidence.

## Introduction

Much of conventional athletic preparation theory is founded on the presumption that specific physical training prescriptions drive predictable biological adaptations. For example, the concept of the repetition maximum (RM) continuum suggests training at specific intensities within specific repetition ranges drives specific performance outcomes [1, 2].

These assumptions are based on a biomedical interpretation of athletic performance. The biomedical model of human health focusses exclusively on biological influences and disregards the potential relevance of psychological, environmental and social influences [3]. Within the sports science literature this view manifests as the implicit belief that training outcomes are fundamentally predictable phenomena due to a mechanistic relationship between physical training and subsequent performance adaptation [4]. An example of this can be seen in a 2018 review where Cunanan et al. state that “the GAS [General Adaptation Syndrome] has proven to be an instructive framework for understanding the *mechanistic process* of providing a training stimulus to induce specific adaptations that result in functional enhancements.” (page 8) [5].

This presumption, that the mechanisms underpinning physical training adaptation are sufficiently well understood to facilitate accurate training prescription, is pervasive within the literature. Existing evidence, however, suggests that an identical training stimulus can lead to differing inter and intra-individual responses with regard to both magnitude and direction. Whilst this fact has been well documented in untrained or recreationally trained individuals, available evidence suggests that the same may be true in athletes [6–10]. Yet, within much of the relevant training-specific published literature, this pervasive inter-individual response, the sources of it and their role are ignored [11].

In part, these individual responses are due to the influence of ‘non-physical factors’, such as psycho-emotional stress, on physical training outcomes which the biomedical model tries to discount [4, 12–14]. Nevertheless, consideration of these factors remains largely absent from standard intervention studies [11, 15]. A suggested reason for this is that, within sports training contexts, such influences are generally considered ‘conditioning’ or confounding factors, rather than fundamental drivers of the primary adaptive signal [5]. These factors then merely need to be controlled for after which the ‘true’ result of the training stimulus can be determined. This conventional perspective, however, appears in direct conflict with contemporary research, emerging across multiple domains. Notably, emerging research suggests that, in the context of biological outcomes, physical and non-physical influences cannot be disentangled [16].

A primary objective of training science is to assist coaches in devising training programmes and processes capable of optimally delivering positive performance outcomes [17]. Yet whether coaches’ perceptions of the core drivers of training adaptation align, or conflict, with the literature remains unknown. Indeed, rarely are coaches’ opinions and perspectives on physical training and performance adaptations reflected in the peer-reviewed literature. However recently, Haugen [18] suggested that coaching practice is often years ahead of sports science in employing critical features of training, subsequently advocating that “the training science community should therefore strive to describe and verify what the best coaches claim to have known for a long time.” (page 1) [18].

In moving to address any potential disconnect between the published literature and practitioner perspectives we surveyed coaches’ views on a range of topics related to the training process. A central objective of this survey was to determine coaches’ perceptions of the core drivers of training-induced performance adaptations. Further, we also sought to determine whether coaches considered non-physical influences to be relevant and important drivers of training outcomes.

## Methods

### Sample Selection and Administration

The survey utilised a purposive convenient sample due to the lack of a centralised coaching database needed for probability sampling. Participation was voluntary, and all those who took part were notified they could withdraw at any point. Due to the fundamental nature of the topics explored within the survey there were limited inclusion criteria of currently working with athletes as a coach, being at least 18 years old and being English literate. With no agreed way to determine sample size for surveys, we established ours on the basis of similar studies leading to a minimum sample of 100 [19, 20]. The survey was available online through Microsoft Forms from November 2021 to February 2022 and was distributed via the authors social media accounts (Twitter and Instagram). To try to limit the potential effects of sampling bias, including the overrepresentation of coaches with strong opinions on certain topics, terms such as ‘general adaptation syndrome’ or ‘periodisation’ did not feature in the advertisement of the survey and appeared only in a limited manner within the survey.

### Study Design and Survey Development

This study used a cross-sectional study approach. After surveying the literature, it was determined that no prior survey asked questions that allowed for the exploration of topics that the authors were interested in. Therefore, it was determined that a new survey would need to be created, with an initial survey being developed by the authors. Once an initial survey was established, recent surveys in the literature were consulted to determine best practice validation [21, 22]. To establish both face and informal content validity, the initial survey was trialled with a small group of experienced coaching practitioners qualified to doctoral level (*n* = 3). The purpose of this was to determine whether the survey properly reflected the relevant literature, and feedback from this process was then used to improve content and clarity. A second round of content analysis combined with piloting was performed on the updated survey specifically focussing on expression of concepts and clarity. For this round, a second group of practitioners representative of the target population (*n* = 7) but not involved in the initial survey validation was used. On the basis of this feedback, final alterations were made to further refine the survey. The final survey was constructed around three distinct topics that ordinarily would be covered in distinctly separate surveys: (1) factors driving physical training adaptation, (2) ‘fundamentals’ of planning training and (3) the predictability of training adaptations. This merging was done due to issues around recruitment and retention of participants. At the outset it was determined that these separate topics would be merged into one survey but then be separated back out for analysis. In part this was also considered necessary as it would not be possible to coherently cover all the topics in a single article. The authors felt that these circumstances met the criteria set out by the APA for separating a single dataset into multiple publications. Within this article a subset of the data referring to questions focussing on the key drivers of physical training adaptation are presented. The questions discussed are available in the supplementary material but are also given below each figure in the results section. Ethical approval for the survey was obtained from the German Sport University Cologne ethics committee.

### Statistical Analyses

Due to the use of convenient sampling, it was decided that we would only present descriptive statistics (in the form of percentage responses, not means or standard deviations) as we could not make any generalisations or inferences to the wider population [23]. Survey responses were exported to Microsoft Excel [24] and anonymised, missing data checks were performed and then the responses were analysed in comparison with the literature. A partial summary of the data is presented in text, with the rest available in the supplementary material.

## Results

### Demographic Information

On closing of the survey, 108 responses had been collected of which 106 agreed to complete the survey. The demographic details of the participants can be seen in Table 1. Participants were predominantly male (92%) with a high level of formal education (60% postgraduate) compared with other studies [20, 25]. The majority (84%) held a coaching qualification. Participants worked with team and/or individual sports at a variety of levels.

**Table 1:** Descriptive characteristics of participants

Gender	%	Age	%	Academic qualification	%	Location	%	Coaching qualification?	%
Male	92	18–34	38	School leaving qualifications	8	UK/Ireland	36	Yes	84
Female	8	35–54	52	Bachelor’s degree	31	Europe (not including the UK or Ireland)	23	No	16
		55–64	8	Master’s degree	49	North America	23		
		65 +	3	Doctoral degree	11	Asia	4		
						South America	4		
						Africa	0		
						Oceania	11		

Years coaching	%	Individual or teams sports	%	Personal participation in sport	%	Level of athlete(s)	%
1–5	20	Team	42	Yes	92	Amateur/recreational	14
6–10	29	Individual	58	No	8	Regional	25
11–15	20					National	29
15 +	31					International	32

### Training and Adaptation

#### Inter-individuality of Adaptation to Training

**Fig. 1:**
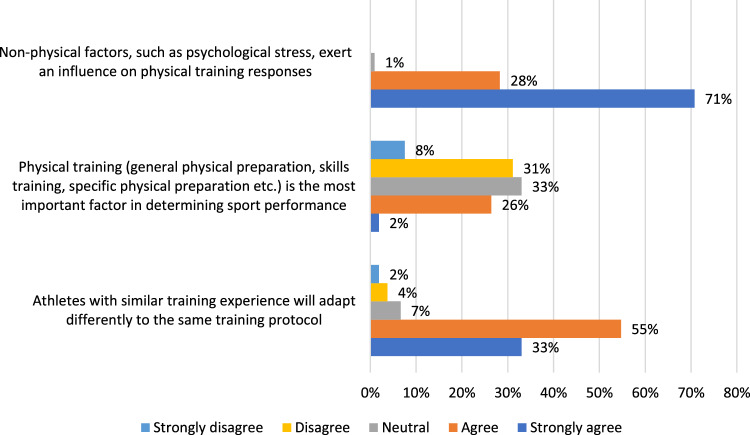
Results to question “Please rate the extent to which you agree or disagree with the following statements”

Figure 1 shows responses to statements regarding inter-individuality of training adaptations. The majority (88%) suggested athletes adapt differently to the same training protocol despite similar training background. More participants disagreed (39%) that physical training was the most important factor in determining sport performance than agreed (28%), though many were neutral (33%). Ninety-nine per cent of respondents indicated that non-physical factors influence physical training response.

#### Modifiers of Adaptation to Training Plan

**Fig. 2:**
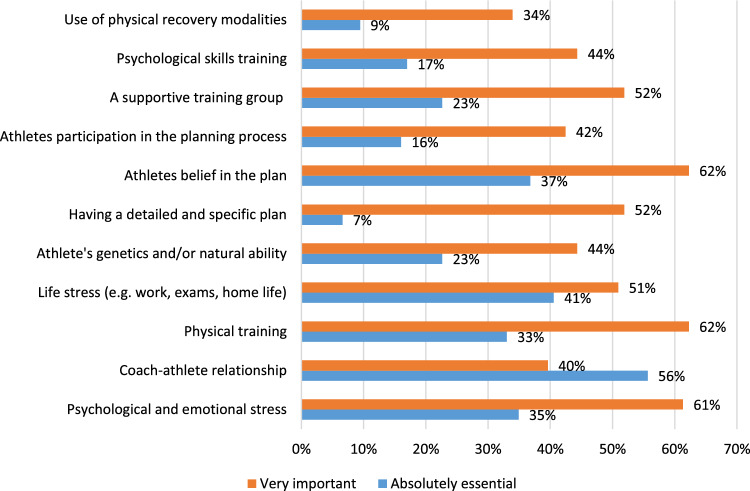
Results to question “How important do you think the following factors are in modifying how well athletes physically adapt to the training plan?”

Data presented in Fig. 2 refer to how important participants considered different factors in modifying an athlete’s ability to physically adapt to a training plan. Only ‘very important’ or ‘absolutely essential’ responses are shown. Other responses can be found in the full dataset (supplementary file 2). Physical training was rated by 95% as either ‘very important’ or ‘absolutely essential’. Coach–athlete relationship had the largest rating of ‘absolutely essential’ (56%), followed by ‘life stress’ (41%), ‘athletes’ belief in the plan’ (37%), ‘psychological and emotional stress’ (35%) and then ‘physical training’ (33%). When looking at the results (supplementary file 2) it would seem that coaches working at different levels seem to weight factors differently, though given the limitations of non-probability sampling we cannot infer the reason for this in the current study.

## Discussion

Given that training research has embraced the use of the biomedical model, and therefore the primacy of the physical over non-physical, our aim was to survey coaches on their opinions of the importance of various factors that can impact the training process. The results show that, whilst many of the coaches opinions were in alignment with the wider scientific literature, they are seemingly at odds with the bulk of training research due the prevailing methodological approach used.

### Coaches Believe Athletes Adapt Differently to the Same Training Protocol

Most participants agreed or strongly agreed that athletes with similar training experiences will respond differently to the same training protocol (Fig. 1). Nevertheless, very few training studies consider or report individual participant data. Indeed most investigations treat individual variation as unavoidable but largely irrelevant noise and seek to minimise its effect through methodological and analytical techniques [26, 27]. However, a recent study, starting from the premise that coaches are likely most interested in applications at an individual level, looked to examine the extent to which group-level results could be generalised to individual athletes [10]. After analysing two seasons worth of load and recovery data (11,055 observations), collected from 82 youth academy football players at a major league club (Eredivisie league), the authors reported different correlations between load and recovery at both a group and individual level. The importance of these results is that they suggest that group-based results do not generalise to individuals. Therefore, employing recommendations based on grouped responses is a suboptimal, or even erroneous, training prescription strategy. As further illustration, Morin et al. [8] demonstrated that sprinters, given the same training protocol, exhibited clear individualisation of performance increases across a range of related metrics, such as 5 and 30 m sprint times. Furthermore individualisation also effected the time for results to be realised post-intervention [8].

The adoption of the biomedical model as the basis for research within training theory means that physical training is the primary source for driving physical adaptation. The effect of this on research is that everything else can be considered a confounding factor that once removed will reveal the “reliability of training effects” [26]. Despite this, in this survey, over a quarter (28%) of coaches agreed that physical training was the most important influence on sport performance, with more (39%) disagreeing. The remaining one-third (33%) were neutral. Furthermore, participants also responded, overwhelmingly (99%), that non-physical factors, such as psychological stress, influence physical training responses. This opinion appears in line with the wider scientific literature illustrating that non-physical factors, such as psychological stress, can dramatically influence general health and physiological outcomes [28]. This poses a problem to the already described approach taken within a large part of the training specific literature as non-physical factors undoubtedly play a role and their effects cannot be separated from physical factors.

### Coaches Believe Non-physical Factors Heavily Influence Training Adaptation

Coaches’ perspectives on the importance of non-physical factors were addressed by asking participants to rate how different factors modified an athlete’s ability to physically adapt to a training plan. Interestingly, four non-physical training influences received the greatest ratings of ‘absolutely essential’, even more than physical training (33%) or genetics factors (23%) [coach–athlete relationship (56%), life stress (41%), athletes’ belief in the plan (37%), psychological and emotional stress (35%)].

Athlete’s belief in the plan was perceived as either ‘absolutely essential’ (37%) or ‘very important’ (62%) by 99% of participants, more than any other factor. Belief in the plan can be thought of as a series of predictions about future events which are formed by an array of factors that include suggestion, observational learning, conditioning and personal relationships like those between coach and athlete [29]. This is sometimes referred to as the placebo effect, which can have very real and powerful effects on a person’s physiology [29].

Of all the factors listed that could modify how well athletes physically adapt to the training plan it was the ‘coach–athlete relationship’ that received the highest rating of ‘absolutely essential’ (56%) by the coaches surveyed. This finding echoes research in medical contexts. As illustration, a recent meta-analysis examining whether patients’ ‘trust’ in their health care professional was associated with health outcomes found that those with higher ratings of trust reported fewer symptoms, demonstrated better adherence to treatments and had higher perceived quality of life [30]. Nevertheless, the perceived relevance of the coach–athlete relationship is not reflected in either conventional training research or theory [31].

Psychological and emotional stress and life stress were considered ‘absolutely essential’ by 35% and 41% of the participants, respectively. The relevance of ‘stress’ has been discussed in the training adaptation literature as well as that of sports injury occurrence and rehabilitation efficacy [13, 32, 33]. It is interesting to note that life stress received a higher rating even though it could be considered a subset of psychological and emotional stress. Regardless, both factors have been shown to have crucial and inseparable impact on the adaptation process [12–14]. An example of this can be seen in Stults-Kolehmainen et al. 2014 study looking at the effect of chronic mental stress on recovery after resistance training [13]. The authors reported that “chronic mental stress has a measurable impact on the rate of functional muscle recovery from strenuous resistance training over a 4-day period. Specifically, higher levels of stress resulted in lower recovery curves and, conversely, lower levels of stress were associated with superior levels of recovery.” (page 8).

Nevertheless, stress outside that of a ‘physical’ nature generally remains ignored within the conventional periodisation literature. This conception of stress, which is grounded in the work of Hans Selye, views these aspects as merely ‘conditioning factors’, a concept which is now outdated. A more modern understanding is that stress (no matter the source) is mediated by the brain as it perceives whether an event is threatening and determines the appropriate behavioural and physiological response [34].

### Coach Education

Notably, coaches’ practical perspectives appear in conflict with the positions of major coach education organisations. As an example, the *Essentials of Strength Training and Conditioning* [1], published by the National Strength and Conditioning Association (NSCA) and “the primary resource to rely on for CSCS [Certified Strength & Conditioning Specialist] exam preparation” [35], acknowledges that various factors exert an influence on training adaptation. Nevertheless, when discussing how to plan a training session or programme, no non-physical factors, such as those highlighted in this survey, are considered.

As illustration, previously the importance of the coach–athlete relationships has been suggested. Jowett and Cockerill, for example, suggested that coach education programmes should not focus only on providing information relevant to physical, technical and tactical skills, but also provide education relating to the fostering of effective relationships with athletes [36]. Nevertheless, the inclusion of non-physical factors within physical training theory, as exemplified by the NSCA’s Essentials of Strength and Conditioning omission of such considerations, remains sparse, at best [1].

### Conducting Training Research

A strict biomedical interpretation of training adaptation, which assumes a mechanistic, and therefore predictable, relationship between conducted training and physiological adaptation, is still pervasive within the literature [2, 11]. Nevertheless, this survey suggests that many practising coaches may hold contrary beliefs given the rated importance of multiple non-physical factors. Therefore, it might be beneficial for future studies to look to other research paradigms that integrate these factors. An example of this could be the biopsychosocial model [3]. It has gained increasing acceptance in medicine due to its acknowledgement of the integrated role of physiological, psychological and social factors in health.

From a methodological perspective given the inter-individual difference in response to training stimuli, studies should report individual responses, either within the study results or as appendices available to the interested reader [37, 38]. Furthermore, to remain practically relevant, training science should collate, consider and, where appropriate, integrate the perspectives of practitioners who plan and deliver athletic training plans.

### Limitations

This is the first global survey to examine coaches’ opinions on a range of factors that drive physical training adaptation. Despite this there are limitations that need to be addressed. As previously mentioned, probability sampling was not used in this study, which carries certain limitations with it such as not being able to make statistical inferences and therefore generalise the results from the current sample to the entire coaching population or specific subpopulations [19, 39]. It is worth noting, though, that the bar is quite high for statistical inferences to be made [40]. However, whilst this is a limitation, the novelty of the survey and its exploratory nature does give insight into some coaches’ perspectives on these important topics. Due to its exploratory nature, further work is needed. With no surveys discussing these topics, the questions were specifically customised. This brings both strengths and weaknesses. Whilst bespoke questions provided novel insights, some questions could be strengthened by an increase in detail or follow-up questions. These would be worthwhile pursuing in future research. Similarly, and inevitably, despite striving for clear expression, some questions may have been misinterpreted [39]. Furthermore, and reflecting a widespread gender bias within performance coaching, only 8% of respondents were female. Finally, this survey was advertised and delivered in English only, and was subsequently unduly biased towards English-speaking participants.

### Recommendations for Future Research

Whether and how coaches integrate non-physical training influences into coaching practice and training plans remains undocumented. Accordingly, investigations detailing coaches’ action and implementation of these considerations, within training contexts, would be beneficial. Similarly, given the neglect of non-physical factors within the physical training literature, it would be interesting to understand how coaches came to hold these perspectives. Finally, the potential relevance of coaches country and/or cultural influences in shaping their beliefs and perspectives around concepts of training adaptation would also be informative [41, 42].

## Conclusion

This is the first survey investigating coaches’ views on the various factors potentially influencing the training adaptation process. Notably, and perhaps surprisingly, amongst coaches surveyed less than a third explicitly rated physical training as the most important factor in determining sports performance. While there was an almost universal belief that non-physical factors exert an influence on physical training response, there was no consensus on the relative importance of each specific non-physical factor.

Importantly, none of the non-physical factors highlighted is typically documented in training and/or periodisation studies. In fact, within the training-specific literature it is difficult to find a study that documents, or even acknowledges, the potential role of non-physical influences in the context of training adaptation. If future training research does not take note of these factors’ role, then the field remains vulnerable to becoming increasingly detached from the coaching community.

Currently, the science seems mired in a strict biomedical conceptualisation of training theory. Many coaches, in contrast, believe non-physical influences effect training adaptations. Nevertheless, this belief remains largely undocumented within the literature and poorly expressed and explained within current training theory.

### Supplementary Information

Below is the link to the electronic supplementary material.

Supplementary file1 (DOCX 14 kb)

Supplementary file2 (XLSX 13 kb)

## Declarations

### Availability of data and material

All data are stored on institutional servers of the corresponding author and are available in the supplementary file.
