# Supplementary material for: Coaches’ Perceptions of Factors Driving Training Adaptation: An International Survey
Source: Sports Med. 2023 Aug 8;53(12):2505–12. doi: 10.1007/s40279-023-01894-1 (PMC10687123; doi:10.1007/s40279-023-01894-1)
Supplement: Supplementary file 1 — Supplementary file1 (DOCX 14 kb) [file 40279_2023_1894_MOESM1_ESM.docx]

| Please rate the extent to which you agree or disagree with the following statements | |
| --- | --- |
| Non-physical factors, such as psychological stress, exert an influence on physical training responses | Strongly disagree  Disagree  Neutral  Agree  Strongly agree |
| Physical training (general physical preparation, skills training, specific physical preparation etc.) is the most important factor in determining sport performance | Strongly disagree  Disagree  Neutral  Agree  Strongly agree |
| Athletes with similar training experience will adapt differently to the same training protocol | Strongly disagree  Disagree  Neutral  Agree  Strongly agree |

| How important do you think the following factors are in modifying how well athletes physically adapt to the training plan? | |
| --- | --- |
| Having a detailed and specific plan | Not important at all  Of little importance  Of average importance  Very important  Absolutely essential |
| Athletes belief in the plan | Not important at all  Of little importance  Of average importance  Very important  Absolutely essential |
| Athletes participation in the planning process | Not important at all  Of little importance  Of average importance  Very important  Absolutely essential |
| Psychological and emotional stress | Not important at all  Of little importance  Of average importance  Very important  Absolutely essential |
| A supportive training group | Not important at all  Of little importance  Of average importance  Very important  Absolutely essential |
| Psychological skills training | Not important at all  Of little importance  Of average importance  Very important  Absolutely essential |
| Coach-athlete relationship | Not important at all  Of little importance  Of average importance  Very important  Absolutely essential |
| Use of physical recovery modalities | Not important at all  Of little importance  Of average importance  Very important  Absolutely essential |
| Physical training | Not important at all  Of little importance  Of average importance  Very important  Absolutely essential |
| Life stress (e.g. work, exams, home life) | Not important at all  Of little importance  Of average importance  Very important  Absolutely essential |
| Athlete's genetics and/or natural ability | Not important at all  Of little importance  Of average importance  Very important  Absolutely essential |
